# Competitive USB-Powered Hand-Held Potentiostat for POC Applications: An HRP Detection Case

**DOI:** 10.3390/s19245388

**Published:** 2019-12-06

**Authors:** Yaiza Montes-Cebrián, Albert Álvarez-Carulla, Gisela Ruiz-Vega, Jordi Colomer-Farrarons, Manel Puig-Vidal, Eva Baldrich, Pere Ll. Miribel-Català

**Affiliations:** 1 Department of Electronics and Biomedical Engineering, Faculty of Physics, Universitat de Barcelona, 08028 Barcelona, Spain; albertalvarez@ub.edu (A.Á.-C.); jcolomerf@ub.edu (J.C.-F.); manel.puig@ub.edu (M.P.-V.); peremiribelcatala@ub.edu (P.L.M.-C.); 2 Diagnostic Nanotools Group, Cibbim-Nanomedicine, Vall Hebron Research Institute (VHIR), Universitat Autònoma de Barcelona, 08035 Barcelona, Spain; gisela.ruiz@vhir.org (G.R.-V.); eva.baldrich@vhir.org (E.B.); 3 CIBER de Bioingeniería, Biomateriales y Nanomedicina (CIBER-BBN), 28029 Madrid, Spain

**Keywords:** horseradish peroxidase (HRP) chronoamperometry, electrochemical biosensor, reconfigurable potentiostat, portable, low-cost electronics, USB-powered

## Abstract

Considerable efforts are made to develop Point-of-Care (POC) diagnostic tests. POC devices have the potential to match or surpass conventional systems regarding time, accuracy, and cost, and they are significantly easier to operate by or close to the patient. This strongly depends on the availability of miniaturized measurement equipment able to provide a fast and sensitive response. This paper presents a low-cost, portable, miniaturized USB-powered potentiostat for electrochemical analysis, which has been designed, fabricated, characterized, and tested against three forms of high-cost commercial equipment. The portable platform has a final size of 10.5 × 5.8 × 2.5 cm, a weight of 41 g, and an approximate manufacturing cost of $85 USD. It includes three main components: the power module which generates a stable voltage and a negative supply, the front-end module that comprises a dual-supply potentiostat, and the back-end module, composed of a microcontroller unit and a LabVIEW-based graphic user interface, granting plug-and-play and easy-to-use operation on any computer. The performance of this prototype was evaluated by detecting chronoamperometrically horseradish peroxidase (HRP), the enzymatic label most widely used in electrochemical biosensors. As will be shown, the miniaturized platform detected HRP at concentrations ranging from 0.01 ng·mL^−1^ to 1 µg·mL^−1^, with results comparable to those obtained with the three commercial electrochemical systems.

## 1. Introduction

Traditionally, diagnostic tests are performed at central laboratories equipped with automated bench-top analyzers that provide highly reproducible and quantitative diagnostic results. Consequently, patients must often wait for long periods before receiving their test results. This circumstance is most common in developing countries and rural areas, where the lack of access to basic diagnostic equipment and trained personnel is an additional challenge [1]. This issue has resulted in an interest to develop Point-of-Care (POC) testing devices in recent years [2]. POC systems are diagnostic instruments that provide rapid results geographically near the patient, even when handled by untrained personnel. Rigorous requirements are set for POC diagnostic systems following the World Health Organization (WHO) ASSURED criteria (Affordable, Sensitive, Specific, User-friendly, Rapid and Robust, Equipment-free and Delivered to end-users) [3]. According to this standard, POC platforms must deliver quick results for early-stage disease detection to enable rapid intervention and improve patient quality of life. The provided results must be accurate, reproducible, and optimally quantitative, and they must be comparable to those obtained by bench-top analyzers at central laboratories. Furthermore, these POC devices must be inexpensive, handheld, or at least portable and easy-to-use, making them usable by non-professional personnel.

Many portable POCs are based on electrochemical detection [4,5,6]. Nowadays, glucose monitoring POC devices (glucometers) are the most widespread miniaturized test systems. Of the main reasons, glucose sensors are inexpensive, easy to produce, small, and easy-to-use [7]. Most glucometers are based on potentiostats, measurement equipment that allow for the studying of oxidation-reduction (redox) reactions taking place in a test solution or at the electrode surface. In this context, the redox activity generates a current proportional to the concentration of the electrochemically active molecules that are being monitored. These systems are suitable in a wide range of applications, such as medical and health care monitoring [8], environmental measurements [9], or construction and characterization of portable biosensors [10], among others. There are different approaches to implement such solutions depending on the requirements of each situation [2], where the budget constraints are one of the main considerations. For instance, conventional bench-top potentiostats designed for research can perform a wide variety of electrochemical analysis. However, they are typically complex, expensive, and tend to be bulky, making them unsuitable for POC applications. In contrast, some research groups have developed miniaturized potentiostat-based single-chip platforms [11,12]. These devices are very small, low-powered, and customizable for specific applications, although the fabrication costs are too elevated for POC implementation.

The use of commercial off-the-shield (COTS) integrated circuits (IC) is an affordable way to miniaturize instrumentation at minimum cost. Some examples based on potentiostats constructed with COTS components can be found in the state-of-the-art for a wide range of applications [13,14,15,16]. For instance, a portable system was developed based on a LMP91000EVM potentiostat (Texas Instruments; Dallas, TX, USA) and a Raspberry Pi 2 Model B microcontroller, which accomplished amperometric detection of progesterone in undiluted saliva making use of disposable immunosensors [17]. A similar COTS-based potentiostat was proposed in [18], which included a wireless module to transmit the measurements to a remote database. The effectiveness of the proposal was assessed by measuring ascorbic acid and comparing the results with those provided by a commercial potentiostat. Another example able to transmit data to a PC or via Bluetooth communication is the portable electrochemical amperometric analyzer proposed in [19]. In this study, the performance and reliability of the platform were validated using an indium tin oxide glass electrode. The low-cost miniaturized potentiostat reported in [20] was also based on an LMP91000 evaluation board, this time assembled to a BeagleBone development board. The system performed cyclic voltammetry (CV) measurements to achieve electrochemical cortisol immunosensing, obtaining a limit of detection of 1 pM of cortisol with a sensitivity of 1.24 µM. In a different approach, Muñoz-Martinez et al. [21] described a system capable of performing electrochemical sensing over system-on-a-chip platforms. To validate the system, they performed diverse electrochemical experiments and a comparison between the COTS system and a commercial potentiostat. Alternatively, smartphones have been exploited as a resource for powering the system, data processing, and Big-Data management [22,23]. One of the most recent advances in the field is the employment of self-powered platforms based on the use of a fuel cell (FC) acting as a power source. In this scenario, the same FC may even act simultaneously as a power source and as a sensor [24]. These solutions use smartphone resources like an audio earphone port [25] or NFC functionality [26].

The main features required for portable electrochemical instrumentation systems are the following: to have a small size, low power consumption, high precision measurements and low fabrication, and maintenance costs. Taking into account these considerations, we have designed a miniaturized, robust, and customizable USB-based system for amperometric detection. The presented system, dubbed AmpStat, is composed of three parts: (a) a connector that houses the disposable amperometric sensor, (b) an embedded electronic system for measurement acquisition, and (c) AmpView, custom software which displays and stores the results. The size of the miniaturized platform is 10.5 × 5.8 × 2.5 cm and it weighs 41 g. The full-custom circuit, which contains the power module and the front-end module, operates a low-voltage condition of 3.6 V and only consumes up to 235 µA. Moreover, a single prototype has an approximate manufacturing cost of $85 USD.

Operation of the proposed system was initially evaluated by detecting amperometrically horseradish peroxidase (HRP). HRP has a particular commercial and medical interest in the molecular biology, medicine, biotechnology, and diagnostic industry fields, as well as broad applicability in life sciences [27]. For instance, HRP is very common in biomedical applications, in which it is used to catalyze hydrogels [28]. On the other hand, HRP and a wide variety of peroxidase enzyme mimetics are extensively employed as electrode modifiers, bioreceptors, and labels to produce enzymatic and sandwich electrochemical biosensors [29,30,31].

As will be shown, the USB-powered prototype developed here provided current measurement from 5 nA to 11 µA in real-time and could be adjusted to register currents up to 3 mA. This allowed detecting HRP at concentrations ranging from 0.01 ng·mL^−1^ to 1 µg·mL^−1^ using screen-printed carbon electrodes (SPCE) and a ready-to-use commercial substrate solution, with results comparable to those obtained with three-commercial potentiostats.

## 2. Materials and Methods

### 2.1. Portable Potentiostat

**Figure 1:**
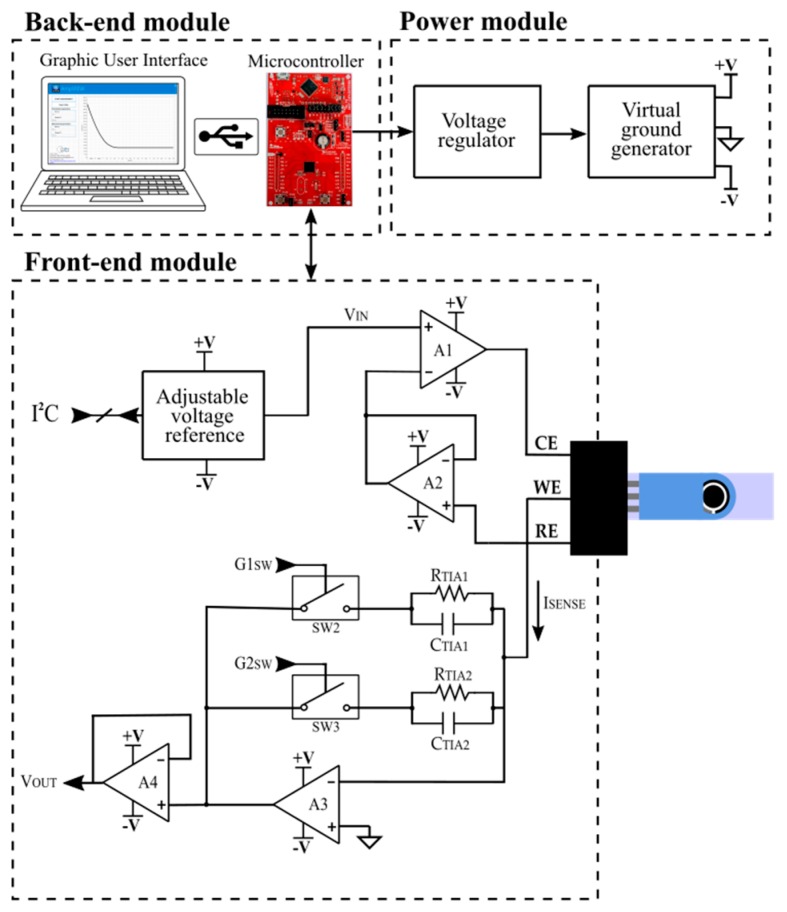
Block diagram of the miniaturized portable potentiostat.

The architecture of the POC system is divided into three parts (Figure 1): (a) a Front-End Module (FEM), which drives the sensor, measures the current provided by the sensor (I_SENSE_) and translates the current into voltage (V_OUT_), (b) a Power Module (PM) that generates a stable voltage and virtual ground, creating a negative supply that allows measuring negative signa, and c) a Back-End Module (BEM), comprised of a microcontroller unit (MCU), which processes the V_OUT_, translates it into a current and sends the result via USB to the computer, and a graphic user interface (GUI), which displays the data on the computer. The block diagram of the system is depicted in Figure 1.

#### 2.1.1. Front-End Module (FEM): Signal Acquisition Module Description

This module performs two tasks: (a) drives the sensor electrodes to the desired voltage (V_IN_) and (b) measures the current provided by the sensor (I_SENSE_), translating it into voltage (V_OUT_) through a Transimpedance Amplifier (TIA) to be read by the MCU.

For V_IN_ generation, we use an adjustable voltage reference based on the AD5321 IC (Analog Devices; Norwood, Massachusetts, USA). This is a 12-bit voltage output Digital-to-Analog Converter (DAC), which has a typical consumption of 120 µA at 3 V and 0.05 µA in power-down mode. The DAC is controlled by the MCU through a 2-wire serial interface, compatible with the I2C protocol.

The designed potentiostat is based on a three-electrode topology. Two operational amplifiers (A1 and A2) polarize the sensor and track V_IN_ to the reference electrode (RE), whose low-input-bias current avoids voltage distortion. An operational amplifier in transimpedance configuration (A3) performs the current readout and translates the current registered between the counter (CE) and the working electrodes (WE) into a voltage signal (V_OUT_) by means of a sensing resistor (R_TIA_). The system includes two gain resistors (R_TIA1_ and R_TIA2_) to measure a wide current range. Two switches (SW_1_ and SW_2_) connect the suitable resistor, depending on the current range to measure. In this way, if the system detects that the measurement is out of range, it automatically changes the R_TIA_ to adjust the measurement circuit to the current range. Two capacitors, C_TIA1_ and C_TIA2_, are connected in parallel with the gain resistors. Both passive components act as a filter, avoiding noise in the corresponding measurement range. Finally, a unity gain buffer amplifier (A4), located between the TIA and the MCU, isolates both parts in order to avoid errors in measurement.

As stated before, the potentiostat circuit translates I_SENSE_ into V_OUT_ through R_TIA_. Both parameters are related according to Equation (1),

V_OUT_ = −R_TIA_ · I_SENSE_ (1)

in which V_OUT_ has a negative value. Accordingly, a dual-supply operational amplifier (positive and negative operation voltage) is required to perform the electrochemical measurement. The operational amplifier used here is a LPV521 (Texas Instruments, Dallas, TX, USA). This nano-power amplifier operates from 1.6 V up to 5.5 V with a typical current consumption of 345 nA. It has a typical offset voltage of 0.1 mV at 1.8 V and a typical bias current of 0.01 pA at 1.8 V. A low-resistance and low-power consumption switch (ADG702; Analog Devices; Norwood, MA, USA) is used to change the current range, which has a typical current consumption of 1 nA and a typical on-resistance of 40 Ω. Moreover, all the resistors used in the system have a 1% tolerance to assure a minimum uncertainty in the measurement.

#### 2.1.2. Back-End Module (BEM): Processing and Display Module Description

The Back-end module is composed of a MCU and a GUI. The MCU used is the MSP430FR5969 LaunchPad development kit. It is a low-cost evaluation module for rapid prototyping, which is based on the MSP430FR5969 microcontroller and includes on-board emulation for programming and debugging. In addition, it is simplified by a 20-pin header, which allows for quick access to General Purpose Input/Output ports (GPIOs), peripherals for communication, Analog-to-Digital Converters (ADC), timers, etc. The MCU controls the DAC, which generates V_IN_ signal through an I^2^C protocol and acquires the V_OUT_ from the FEM by means of the ADC. It also activates the suitable switch, which sets the best R_TIA_ value assuring the maximum measurement range. Furthermore, it processes the obtained data, translating it into current and sending it to the computer via USB port.

**Figure 2:**
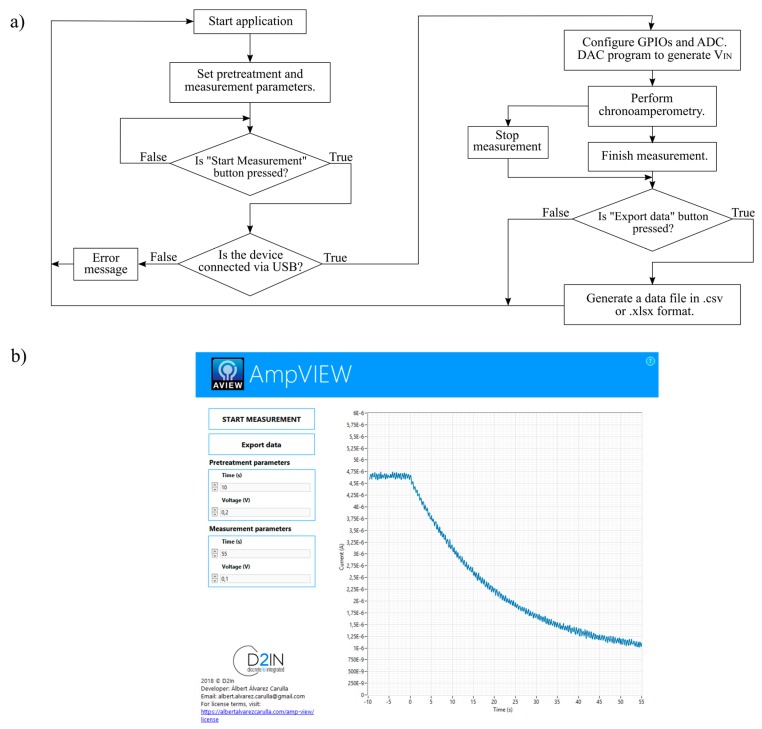
(**a**) Diagram flow of the designed program. (**b**) Chronoamperometry curve measured with AmpVIEW.

The full-custom GUI, AmpVIEW, is developed using LabVIEW (National Instruments; Austin, TX, USA), a development software for visual language programming. AmpVIEW controls the system, including the measurement time and applied voltage and, at the same time, it registers and displays the chronoamperometry measurement in real-time. Figure 2 summarizes the procedure followed to perform measurements and a picture of the GUI. Once the program has been turned on, the user must set the pretreatment and measurement parameters (time and sensor’s polarization voltage) and must press the “Start Measurement” button. An error message appears on the display when the device has not been connected to the computer and the process ends. In the case of having the device connected, the MCU configures the GPIOs and the ADCs ports, it controls the DAC through the I^2^C protocol and it starts the chronoamperometry. While the FEM drives the sensor, the MCU acquires the V_OUT_ signal and it sends the result via USB to the GUI, which displays the data in real-time. When the measurement is finished, the user can save the data in a file pressing the “Export Data” button, which generates a csv or xlsx file with the chronoamperometry data. In addition, the measurement can be aborted at any moment by pressing the “Stop Measurement” button.

#### 2.1.3. Power Module (PM): Power Supply Module Description

The full-custom circuit board is connected to the MCU board, which operates with the power provided by the computer USB port. The MCU board provides 3.6 V (VMCU) to the full-custom circuit that contains the FEM and the PM. In order to avoid variations in the power supply, the V_MCU_ is sent to the AP3330 Low Dropout linear Regulator (LDO), which provides an output with a stable and regulated voltage of 3.6 V (V_PM_). As stated in the previous section, it is necessary to have dual supply operational amplifiers to measure the negative voltage obtained at the potentiostat’s output (V_OUT_).

To generate the negative voltage supply, we created a virtual ground with a low quiescent current LDO (ADP125 IC; Analog Devices; Norwood, Massachusetts, USA). This provides an output voltage of 3 V that is set as a virtual ground, by creating a non-symmetrical power supply of 0.6 V (+V) and −3 V (−V), allowing a wide negative measurement range. The ADP125 has a quiescent current of 45 µA with no-load and a maximum quiescent current of 210 µA in case of maximum load (500 mA).

### 2.2. Electrochemical Measurements

#### 2.2.1. Electrochemical HRP Detection Procedure

The chronoamperometric detection of HRP (Ref. P6782, Sigma Aldrich, San Luis, MO, USA) was performed in parallel using the full-custom AmpStat potentiostat and three commercial potentiostats. Disposable screen-printed carbon electrodes (SPCE; Ref. DRP-110; Dropsens; Llanera, Spain) were employed for this purpose, each one featuring a 4-mm carbon working electrode, a carbon counter electrode, and a silver pseudo-reference electrode. Before they were used, SPCE were rinsed with ethanol 70% and water, dried under airflow and characterized by CV in 0.1 M KCl, 1 mM K_4_Fe(CN)_6_.

For detection, HRP was diluted serially in miliQ water, obtaining eight final HRP concentrations (0.01, 1, 10, 25, 50, 100, 500, and 1000 ng·mL^−1^) and a negative control without HRP. The measurement equipment was turned on to register current at 0.00 V vs. the Ag pseudo-reference. Then, the SPCE was plugged and 45 µL of ready-to-use 3,3′,5,5′-Tetramethylbenzidine (TMB) Liquid Substrate System (Sigma Aldrich, Ref. T0440, which contains a mixture of TMB and H_2_O_2_ in a buffer of undisclosed composition) were pipetted onto the electrodes. Next, 5 µL of HRP were added and the enzymatic reaction proceeded while the current was registered for 300 s more.

#### 2.2.2. Data Analysis

Data shown in the graphs correspond to the averages of no less than three independent replicates, each one obtained using a new SPCE, and the error bars show their standard deviation (SD). The assay limits of detection (LOQ) and quantification (LOQ) were calculated using the average of the blanks plus 3 and 10 times their SD, respectively. The variability was estimated in terms of the variation coefficient (%CV = (SD/mean) × 100). The sensitivity corresponded to the slope of the linear range of the assay and the signal-to-noise ratio (SNR) was the signal registered for each HRP concentration divided by the averaged signal of the blanks.

## 3. Results and Discussion

In this work, we report the design, production, and analytical evaluation of a USB-powered portable potentiostat. The AmpStat prototype was designed to detect amperometrically the activity of HRP, an enzyme widely used for the detection of electrochemical biosensors, although it could be easily adapted for other applications.

### 3.1. Test and Calibration of the Electronic Platform

**Figure 3:**
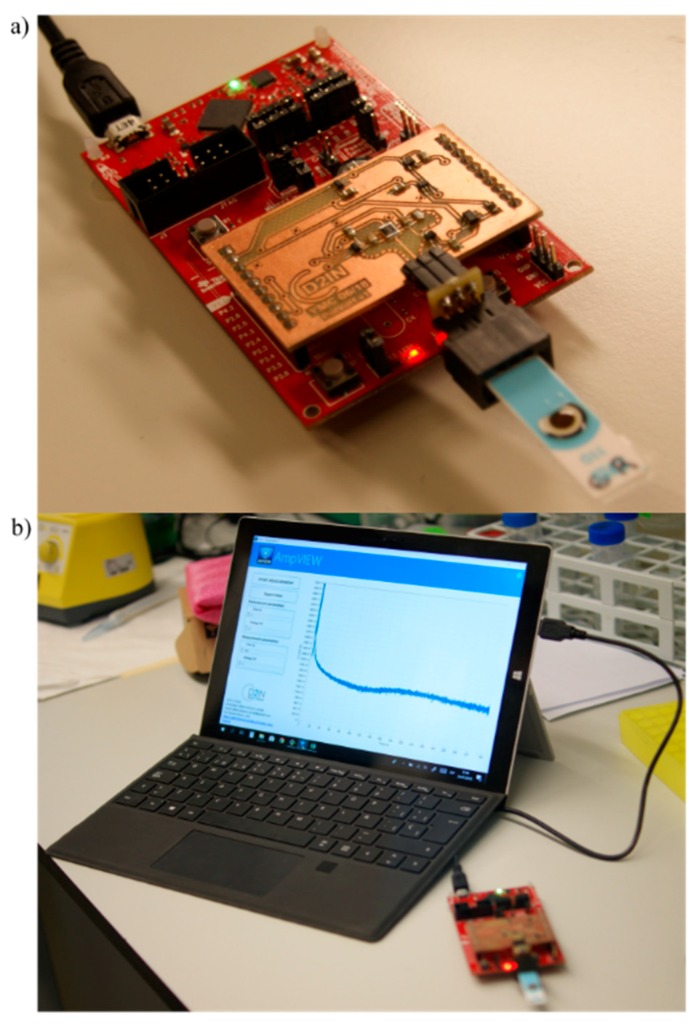
Picture of the prototype developed. (**a**) AmpStat potentiostat composed by the full-custom PCB (copper board) and the MCU board (red board). (**b**) AmpVIEW software registering an amperometry in real time.

Figure 3 shows the final device composed by the full-custom Printed Circuit Board (PCB) connected to the MCU board (Figure 3a) and the control AmpVIEW software (Figure 3b). The whole system measures 10.5 × 5.8 × 2.5 cm and it weighs 41 g. The PCB, which contains the FEM and the PM, is connected to the MCU board through a 20-pin header. Since the FEM circuit measures low currents, we implemented a guard ring used to protect high-impedance circuit nodes from surface leakage currents. It is a copper ring driven by a low-impedance source to the same voltage as the high impedance node.

We designed and simulated the electronic circuit with Multisim program (National Instruments; Austin, TX, USA), and we made the PCB layout using Ultiboard software (National Instruments; Austin, TX, USA). The MCU was programmed with Code Composer Studio (Texas Instruments; Dallas, TX, USA), an integrated development environment (IDE) to develop applications for Texas Instruments embedded processors.

The electronics characterization and a preliminary study towards the analytical validation of the platform were carried out with a Source Measurement Unit (SMU) B2962A (Keysight Technology; Santa Rosa, CA, USA) and an oscilloscope MSO-X 3034A (Agilent Technologies; Santa Clara, CA, USA). This allowed analyzing the AmpStat performance and deviation by measuring the I_SENSE_ current. For this purpose, the SMU was connected to the electronics platform and was programmed to apply a ramp of current from 0 µA to 11.2 µA (maximum current that the system can measure) while I_SENSE_ was measured. The maximum deviation between the current measured and the current applied was of 4.26%. The AmpStat potentiostat, composed by the PCB and MCU board, consumes 6.45 mA at 5 V, although the PCB, which contains the FEM and PM, only consumes 235 µA, and can work with voltages of 3.6 V. The difference between consumptions is due to the MCU board, which contains many functionalities that increase prototype consumption. The developed system is able to measure currents up to 11.2 µA with resolution 0.13 nA. However, it can be configured to measure currents up to 3 mA. Furthermore, the system can polarize the sensor electrodes to voltages up to 3.6 V.

Compared to other works, AmpStat had a broader current range and resolution, while still maintaining comparable operation voltage [32,33]. The measurement deviation of AmpStAT was lower than the proposed by [34], which was the only work we could find in which this parameter was discussed. Although the potentiostat reported in [11] consumes less power, it is a fully-integrated chip designed in a 0.35-μm bulk-CMOS technology and can measure a more limited current range.

### 3.2. System Verification by HRP Electrochemical Detection

**Figure 4:**
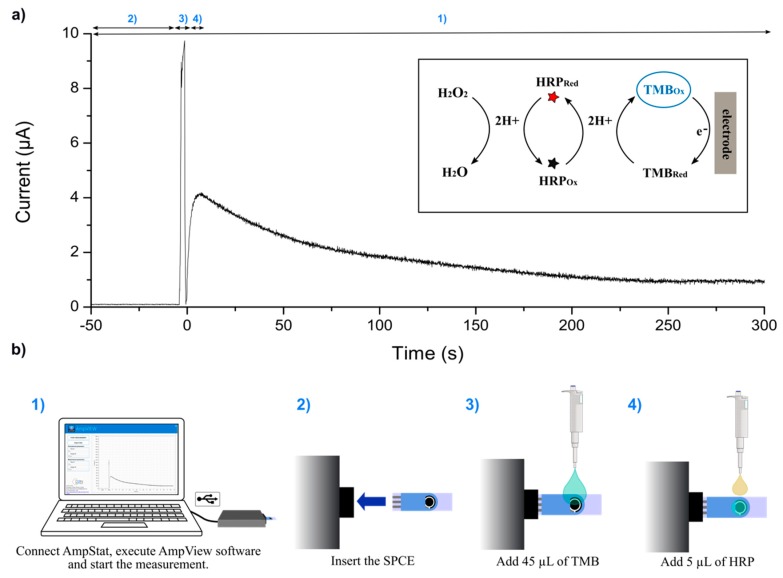
(**a**) Example of a chronoamperometry registered for 500 ng·mL^−1^ of HRP. (**b**) The procedure followed for current measurement: (1) First, the AmpStat potentiostat was connected via USB to the computer, the AmpView software executed, and the “Start” button clicked on; (2) the SPCE was plugged to the connector, and (3) 45 µL of TMB were added; then, to begin the enzymatic reaction, (4) 5 µL of HRP were pipetted on the electrodes, and the current was registered during 300 s; finally, the “Stop” button was pressed and the data saved.

The equipment was tested by detecting HRP concentrations ranging from 0.01 ng·mL^−1^ to 1000 ng·mL^−1^, as well as a negative control without HRP. HPR was detected by monitoring its activity using a commercial ready-to-use substrate solution that contained TMB and H_2_O_2_. In this system, HRP catalyzed the reduction of H_2_O_2_ coupled to the oxidation of TMB. The resulting oxidized TMB was then reduced at the surface of a SPCE, which registered a reduction current that was proportional to the amount of HRP present in solution (insert in Figure 4a). The AmpStat prototype was able to apply voltages up to 3.6 V between the WE and the RE. However, sensor characterization revealed that the best potential for this application was 0.00 V vs. the Ag pseudo-reference, and these were the measurement conditions used here.

Figure 4 shows an example of chronoamperometric detection for 500 ng·mL^−1^ of HRP, and the procedure followed to perform each detection. First, we connected the AmpStat to the computer, executed the AmpView program, and pressed the “Start” button to record the current signal (Figure 4b(1)). The software started showing the chronoamperometry in real-time on the computer display. Then, we plugged the SPCE to the potentiostat (Figure 4b(2)) and pipetted 45 µL of TMB onto the electrodes, which produced a transient current fluctuation (Figure 4b(3)). We then added 5 µL of HRP (Figure 4b(4)), and allowed the enzymatic reaction to take place while the current was registered for 300 s. Finally, when the measurement ended, or the “Stop” button was pressed, data was saved in csv or xlsx format.

**Figure 5:**
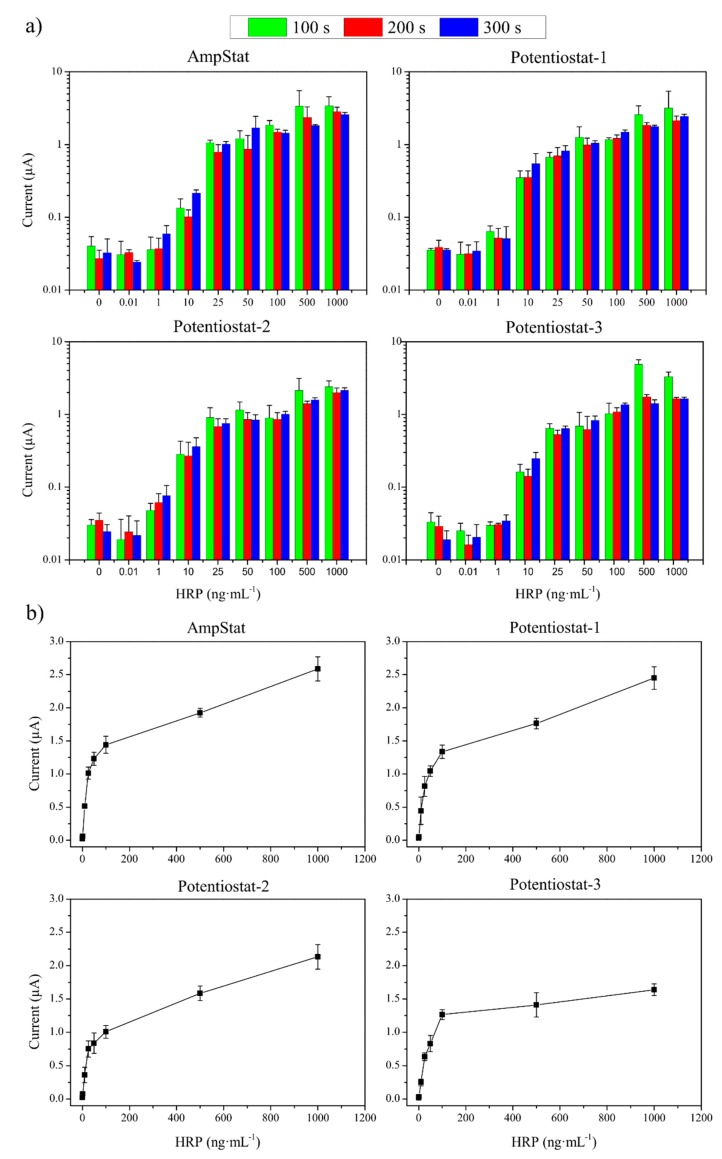
(**a**) Currents recorded by the four potentiostats used for HRP concentrations ranging from 0 to 1000 ng·mL^−1^ after 100 s, 200 s and 300 s of reaction with the substrate. (**b**) Currents registered after 300 s of reaction with the substrate (n = 3).

Figure 5a shows a histogram summarizing the currents registered over time for increasing HRP concentrations and employing alternatively the customized AmpStat and the three commercial potentiostats used in parallel as the reference standards. As can be seen, the equipment generated comparable results. In general, the enzymatic reaction was fast, and the highest currents were registered over the first 100 s for HRP concentrations above 25 ng·mL^−1^. In contrast, detection of lower HRP concentrations improved for longer measurement times. Additionally, the fourth equipment displayed the highest reproducibility at 300 s, when current stabilization had been reached. In all cases, the currents registered ranged 20–50 nA for the blanks, increased proportionally to HRP concentration up to 100 ng·mL^−1^, and increased to a lesser extent or plateaued for higher HRP contents when the reaction was substrate-limited (Figure 5b).

**Figure 6:**
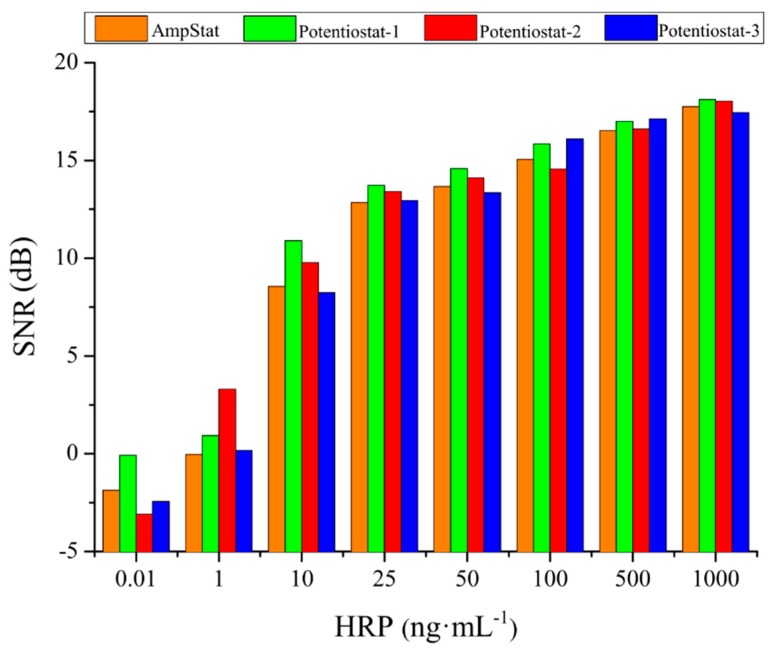
SNR of the four potentiostats for HRP concentrations ranging from 0.1 to 1000 ng mL^−1^.

Among the three commercial equipment that were studied, the potentiostat-3 was the one that measured the lowest currents for all HRP concentrations, but also presented the smallest data dispersion. On the other hand, the potentiostat-2 registered the highest currents and the most stable and reproducible background noise in the blanks (see the currents registered over time for the blanks in Figure 5a). Interestingly, the AmpStat portable system developed here measured similar currents and comparable result dispersion than the potentiostat-2 (%CV ranging of 4.7–21% in both cases). Nevertheless, the AmpStat displayed more variable blank measures. This negatively affected the assay SNR, which was calculated by using the blank values (Figure 6).

Table 1 summarizes the figures of merit calculated for the HRP detection assay, carried out with the four electrochemical systems. The four potentiostats displayed comparable LOD and LOQ in terms of minimal current measurable and quantifiable, respectively. However, in terms of HRP concentration, both LOD and LOQ were higher (and thus worse) if HRP was measured using the potentiostat-3. The customized AmpStat potentiostat designed here exhibited an LOD of 0.83 ng·mL^−1^ and an LOQ of 1.52 ng·mL^−1^ of HRP, which were values only slightly higher than those provided by the potentiostat-1 and the potentiostat-2 (0.52–0.56 ng·mL^−1^ and 1.16–1.61 ng·mL^−1^, respectively). Additionally, assay sensitivity, calculated as the slope of the corresponding assay linear range (spanning from 0.01 ng·mL^−1^ to 50 ng·mL^−1^), was comparable for all three equipment. These parameters were reasonably accurate, taking into account that the AmpStat is a homemade prototype with an approximate manufacturing cost of $85 USD.

## 4. Conclusions

In this paper, we demonstrated experimentally the performance of AmpStat, a full-custom low-cost potentiostat. We described in detail the design, production, and preliminary analytical evaluation of this portable high-performance prototype, which is entirely powered and controlled by USB and includes a user-friendly interface that makes it plug-and-play and easy-to-use.

The potentiostat is composed of three blocks: the PM, which generates a stable voltage and a virtual ground, the potentiostat-based FEM, and the BEM, which includes an MCU and the GUI. Furthermore, we developed a full-custom software, called AmpVIEW, which controls the system, presents the chronoamperometry in real-time on a computer display, and allows saving the data in different file formats. The low-cost platform consumes 6.45 mA at 3.6 V. However, the full-custom designed PCB, which contains the instrumentation, only consumes 235 µA. Furthermore, the developed potentiostat can drive the sensor electrodes to voltages up to 3.6 V and measure currents up to 11.2 µA, although it can be adjusted to measure a maximum current of 3 mA.

We confirmed the efficacy of the AmpStat prototype by detecting HRP in a concentration range from 0.01 ng·mL^−1^ to 1 µg·mL^−1^. As has been shown, the results obtained with the AmpStat were comparable to those obtained using three commercial electrochemical systems that were significantly more expensive. Our equipment displayed LOD of 0.83 ng·mL^−1^, LOQ of 1.52 ng·mL^−1^, and sensitivity of 0.0305 µA·mL·ng^−1^.

Furthermore, this platform could be additionally optimized in the future to produce a tailored compact system. For instance, size reduction could be achieved by creating a full-custom board with an integrated microcontroller unit. The software could also be upgraded to perform current averaging within defined measurement time ranges and/or interpolation in preloaded calibration plots. Also, the system could integrate additional functionalities based on mobile resources. For example, the incorporation of a Bluetooth module to connect the system to a smartphone and a rechargeable battery would facilitate the employment in POC testing, resulting in a more compact and flexible electrochemical detection solution.

## Figures and Tables

**Table 1:** Comparison of the figures of merit of the HRP detection assay, carried out with the four tested potentiostats.

		AmpStat	Potentiostat-1	Potentiostat-2	Poteniostat-3
LOD					
	Current (µA)	0.05	0.05	0.04	0.04
	Concentration (ng·mL^−1^)	0.83	0.52	0.56	1.27
LOQ					
	Current (µA)	0.07	0.08	0.07	0.06
	Concentration (ng·mL^−1^)	1.52	1.16	1.61	2.89
	Sensitivity (ng·mL^−1^·µA^−1^)	0.0328	0.0326	0.0305	0.0219
	Weight (g)	41	1600	480	5433
	Dimensions (cm)	10.5 × 5.8 × 2.5	22.2 × 20.5 × 7.5	13.2 × 10.0 × 3.6	360.7 × 233.7 × 116.9
	Cost (USD)	85	11,013	4087	16,000
